# Stressors related to the Covid-19 pandemic, climate change, and the Ukraine crisis, and their impact on stress symptoms in Germany: analysis of cross-sectional survey data

**DOI:** 10.1186/s12889-022-14682-9

**Published:** 2022-11-30

**Authors:** Roland Weierstall-Pust, Thomas Schnell, Philipp Heßmann, Michael Feld, Max Höfer, Anna Plate, Matthias J. Müller

**Affiliations:** 1 Oberberg Clinics Group, Hausvogteiplatz 10, Berlin, 10117 Germany; 2 Medical School Hamburg, Hamburg, Germany; 3 Private Practice for General and Sleep Medicine, Frechen, Germany; 4 Höfermedia Communication Agency, Berlin, Germany; 5 Justus Liebig University Gießen, Gießen, Germany

**Keywords:** Covid-19, Ukraine war, Climate change, Covert stress effects, Physical stress response, Psychological stress response, Perceived stress, Representative cross-sectional study, Germany

## Abstract

**Background:**

Climate change, the Covid-19 pandemic, and the Ukraine crisis are considered unprecedented global stressors, potentially associated with serious health consequences. However, simultaneous effects of these stressors are not yet understood, making it difficult to evaluate their relative contribution to the population burden and potential future manifestations in clinically significant psychiatric disorders. This study aimed at disentangling the relative contribution of the three stressor groups on current sub-clinical stress symptoms.

**Methods:**

A cross-sectional, representative survey study was conducted two months after the outbreak of the Ukraine war in Germany. Proportional quota sampling was applied for age, gender, income, and regional characteristics. Data were recruited by means of an online survey. 3094 data sets (1560 females) were included. Age ranged from 18–89 (*M*: 50.4 years; *SD*: 17.2). The Subclinical Stress Questionnaire (SSQ-25) served as main outcome measure. In collaboration with a professional media agency, 20 items were generated to capture salient population stressors. A three-factor exploratory structural equation model confirmed the appropriateness of this scale.

**Results:**

(1) Differences in subjective rankings revealed that stressors related to the Ukraine crisis were rated as most worrying, followed by climate change, and the Covid-19 pandemic (Generalized-Linear-Model: Epsilon = .97; *F*(1.94, 6001.14) = 1026.12, *p* < .001; *η*_*p*_^2^ = .25). (2) In a linear regression model (*R*^*2*^ = .39), Covid-19 pandemic stressors were the only meaningful predictors for current ill-health (standardized *β* = .48). Ukraine crisis did not predict stress symptom profiles in the present sample. (3) Older and male individuals report less and/or less severe stress symptoms, although effect sizes were small (range: *η*^*2*^ .11—.21). An older age also reduced the impact of Covid-19 stressors.

**Conclusions:**

Researchers from the health sciences must consider overlapping effects from population stressors. Although the Ukraine crisis and climate change mark salient stressors, including economic threats, the Covid-19 pandemic still has a profound effect on ill-health and must be considered as a relevant factor in future manifestations of psychiatric and associated health consequences.

## Background

Population stressors, i.e. stressors that may leave lasting imprints on an entire population and often result in serious health consequences, have well been investigated in former crises and conflict regions globally. Stressors include natural disasters [1, 2], outbreaks of infectious diseases [3, 4], or violent crises [5, 6]. Lazarus and Folkman [7] state that stress occurs when the demands of the environment exceed the resources available to cope with a threatening situation. Most etiological risk models for mental disorders suggest that chronic stress, as well as highly salient stressors, that are characteristic for times of crisis, can deteriorate mental health [8] and have both, short- and long-term adverse effects [9]. Psychiatric research, in particular in the field of psychotraumatology, consistently demonstrates that population stressors may manifest in clinically significant disorders even years after exposure [10, 11]. Outside Europe, researchers have started to disentangle the mental health effects of different population stressors in societies that have faced more than one adverse population stressor: The reciprocal effects of abduction and HIV infections in former Ugandan child soldiers [12] or the interplay between displacement and poverty in conflict-affected populations in Sudan [13] are just few examples that stress the necessity to differentiate between stressors simultaneously affecting populations and recruit distinct and effective countermeasures. With an increasing awareness for the adverse mental health consequences of climate change, the global health effects of the Covid-19 pandemic, and the recent outbreak of the Ukraine crisis, western European currently face a series of significant population stressors. But what are their differential effects on mental health?

(1) Climate change as a direct cause for mental disorders, for example due to exposure with natural disasters, has received considerable attention in the scientific literature [14, 15]. In the past years, the existential threat, as well as psychological distress and anxiety about climate associated future crises have gained attention in the literature, focusing on the perceived threat -beyond actual natural disasters- as a risk factor for mental health issues [16, 17]. (2) For the case of Covid-19, previous epidemics already indicated that mental health can be severely affected [18, 19]. A large study showed that COVID-19 stressors were associated with psychopathological symptoms in more than half of the respondents [20]. Stress occurred through insufficient information, fear of infection, and duration of quarantine [21–23]. Far-reaching consequences of a COVID-19-associated lockdown affecting daily routines and social interactions have also been linked to considerable mental health issues [24]. These factors may generate a feeling of loss of control and reduce the people’s sense of coherence, both associated with psychopathological reactions [25]: the feeling of security taken for granted in everyday life and the anticipated predictability of the future appear increasingly uncertain. In addition, research on consequences of the COVID pandemic identified vulnerable subgroups, such as young people, women, and employees in helping professions [26, 27]. (3) For the burden related to the recent Ukraine crisis, researchers address the direct health consequences for the Ukrainian society [28, 29], as well as challenges for countries hosting refugees [30, 31]. To the best of the authors’ knowledge, no research has been conducted yet that acknowledges mental health consequences of perceived threats in neighboring countries due to direct and indirect consequences of the war, although research from other conflict zones demonstrates adverse consequences of enduring tensions [32, 33].

A distinctive feature of the three recent population stressors currently affecting western European societies is their timely overlap and research so far is often limited to the study of single effects. In addition, these events may require social and individual adaptation efforts that go far beyond the usual extent of individual coping capacities. In particular, the Covid-related measures led to a temporary shutdown of the usual public life in many countries. Findings from standard stress research may fall short in explaining effects of these current stressors, when stressor groups are analyzed independently. Recent research has already started to point out the significance of disentangling the differential effects population stressors may have simultaneously. In recent studies on youth and young adults in the United Kingdom and Germany, it could be demonstrated that the Covid-19 pandemic and climate change are both associated with distress but evoke different emotional reponses [34, 35]. Likewise, it could be demonstrated in another study in UK residents on worries related to the Covid-19 pandemic and climate change that although people have no finite pool of worries, different stressor groups may have differential behavioral effects [36] Other studies, for example from China, could demonstrate that worries in relation to different stressor groups can even fuel each other [37] Thus, the analysis of distinct effects of different stressor groups on population health can be considered topical, as such global population stressors may leave lasting imprints on mental health at least in Western or high-income countries.

The study aimed to (1) compare the perceived worries about the three population stressors, and (2) disentangle their relationship with current subclinical stress symptoms in a representative German community sample.

## Methods

### Samples and procedures

Respondents were 3101 adults from a representative, cross-sectional community sample in Germany. Sampling took place in the last week of April and first week of May 2022, about two months after the outbreak of the Ukraine crisis, and immediately after the second peak of Corona wave five in Germany, in February and April 2022 (Variant of Concern: Omikron BA.2; phase 7b [38]). Seven respondents indicated a diverse gender and could not be considered in subgroup analyses for power reasons. The remaining *n* = 3094 data sets included 1560 females and 1534 males. Participants’ age ranged from 18–89 years (*M* = 50.4; *SD* = 17.2 years). Table 1*displays sample characteristics*.

**Table 1:** Sociodemographic data (No. (%))

Factor	females (*n* = 1.560)	males (n = 1.534)	total (*n* = 3.094)	test statistics
***age group***				
18–29	244 (15.6%)	247 (16.1%)	491 (15.9%)	*Chi*^2^ (5) = 3.64, *p* = .603
30–39	220 (14.1%)	221 (14.4%)	441 (14.3%	
40–49	252 (16.2%)	257 (16.8%)	509 (16.5%)	
50–59	320 (20.5%)	279 (18.2%)	599 (19.4%)	
60–69	246 (15.8%)	233 (15.2%)	479 (15.5%)	
70–99	278 (17.8%)	197 (19.4%)	575 (18.6%)	
***net income group (month)***				
0–1.000€	330 (21.2%)	220 (14.3%)	550 (17.8%)	*Chi*^2^ (5) = 158.84, *p* < .001
1.001—1.999€	519 (33.3%)	437 (28.5%)	956 (30.9%)	
2.000—2.499€	160 (10.3%)	246 (16.0%)	405 (13.1%)	
2.500—3.499€	102 (6.5%)	201 (13.1%)	303 (9,8%)	
> 3.500€	75 (4.8%)	191 (12.5%)	266 (8,6%)	
no answer	374 (24.0%)	240 (15.6%)	614 (19.8%)	
***federal state***				*Chi*^2^ (15) = 10.87, *p* = .762
Baden Württemberg	206 (13.2%)	190 (12.4%)	396 (12.8%)	
Bavaria	238 (15.3%)	237 (15.4%)	475 (15.4%)	
Berlin	72 (4.6%)	77 (5.0%)	149 (4.8%)	
Brandenburg	49 (3.1%)	46 (3.0%)	95 (3.1%)	
Bremen	14 (0.9%)	14 (0.9%)	28 (0.9%)	
Hamburg	41 (2.6%)	45 (2.9%)	86 (2.8%)	
Hesse	113 (7.2%)	112 (7.3%)	225 (7.3%)	
Mecklenburg-Vorpommern	36 (2.2%)	25 (1.6%)	60 (1.9%)	
Lower Saxony	146 (9.4%)	153 (10.0%)	299 (9.7%)	
North Rhine Westphalia	319 (20.4%)	333 (21.75)	652 (21.1%)	
Rhineland Palatinate	90 (5.8%)	62 (4.0%)	152 (4.9%)	
Saarland	22 (1.4%)	14 (0.9%)	36 (1.2%)	
Saxony	74 (4.7%)	83 (5.4%)	157 (5.1%)	
Saxony Anhalt	42 (2.7%)	46 (3.0%)	88 (2.8%)	
Schleswig Holstein	54 (3.5%)	56 (3.7%)	110 (3.6%)	
Thuringia	45 (2.9%)	41 /2.7%)	86 (2.8%)	

*Abbreviation*: *p* = level of significance

Although quota sampling considered equal distributions, respondents were free to report their net income for consideration in the data analysis. Therefore, significant differences for income across female and male respondents were found. Inclusion criteria were fluency in German, access to Internet and fully written informed consent. No further exclusion criteria were applied. The Ethics Committee of the Medical School Hamburg provided ethical clearance. The KANTAR agency recruited data by means of an online survey. Proportional quota sampling was applied for age, sex, income, and regional characteristics for a representative weighting method. A forced answering option was chosen so that missing data was not an issue. All respondents received financial compensation.

### Measures

#### Assessment of subclinical stress symptoms

The Subclinical Stress Questionnaire (SSQ-25) [39, 40] is a validated 25-item self-rating instrument (5-point Likert rating scale; 0 = not at all; 4 = extremely). It contains two subscales (psychological stress symptoms (15 items); physiological stress symptoms (10 items)). A sum-score as well as the two subscale scores were computed. In the present sample, good internal consistency of Cronbach's alpha = 0.96 for the sum score was found (psychological subscale: 0.96; physiological subscale: 0.90).

#### Assessment of sub-clinical stress symptoms related to stressor groups

Further 20 items related to the population stressors were generated (climate change stressors: 5 items: Covid-19 pandemic stressors: 8 items; Ukraine crisis stressors: 7 items). Items were generated on basis of current population fears discussed in the public media with support of media experts, aiming to capture the most relevant stressors. Climate change stressors included for example worries about natural disasters, adverse consequences for future generations, or an increase in geopolitical conflicts (sample items: “I’m worried that the number of natural disasters in Germany will increase”; “I’m worried that future generations might suffer from the consequences of climate change”; “I’m worried that climate change will lead to an increase in geopolitical conflicts”). Covid-19 pandemic stressors covered worries about the respondent’s own health, the health of family members, restrictions in daily life, economic consequences, or social erosion (sample items: “Due to the Covid-19 pandemic, I’m worried about my own health”; “Due to the Covid-19 pandemic, I’m concerned about my own economic situation”; “I’m worried about social erosion in Germany, due to the Pandemic”). The stressors related to the Ukraine crisis covered worries about the fate of the Ukrainian people, economic consequences (including inflation), or an extension of the war (sample items: “I’m worried about the Ukranian people”, “I’m worried about the economic consequences of the war, for example an increase in energy costs”; “I’m worried that the war might have an impact on geopolitical safety, for example in terms of a third world war”). Current subclinical stress symptoms were rated on a 5-point Likert scale (0 = not at all; 4 = extremely). Satisfying internal consistency was achieved, with Cronbach's alpha of 0.88, 0.90, and 0.89 for the three scales. Exploratory Structure Equation Model (ESEM) [41, 42] was applied using a three-factor model to prove the validity and distinctiveness of the three scales. Assessment of overall model fit was based on fit indices, including Root Mean Square Error of Approximation (RMSEA), Standardized Root Mean Square Residuals (SRMR), Tucker-Lewis reliability Index (TLI) and Comparative Fit Index (CFI). According to Hu & Bentler [43], values < 0.08 for RMSEA and SRMR represent good model fit. Hoyle [44] and Byrne [45] propose values between 0.90-0.95 for CFI and TLI, also representing good model fit. The proposed factor structure achieved satisfying fits (RMSEA = 0.07; SRMR = 0.03.; CFI = 0.95; TLI = 0.92). Due to the unequal number of items per scale, scale item means were calculated. Additionally, scale means for the stressor groups were ranked within individuals.

### Statistical analysis

Descriptive sample characteristics were calculated. Non-parametric zero-order-correlations between SSQ-25 sum score and subscales, stressor scales, and age. For gender-differences, non-parametric comparisons (U-test) were performed. Scale rankings were compared applying Friedman test statistics for paired samples. Pair-wise comparisons utilized Wilcoxon tests. Multivariate linear regression analyses (backward exclusion) were calculated with the sum score of SSQ-25 as dependent variable, and the following predictors: Age, gender, climate change scale, Covid-19 pandemic scale, Ukraine crisis scale. Further, two-way mean-centered interactions between age, sex, and stressor scales were added to the regression model. Calculation of Cook’s distances and the variance inflation factors (VIF) were applied analyzing outliers and multi-collinearity. Analyses were performed using SPSS and AMOS 28 for windows, as well as R statistics and MPlus [45, 46]. Effect sizes were calculated using G*power 3.1 [47]. Level of significance was set to 0.05.

## Results

### Inter-correlations between the main outcome variables

Zero-order correlations were calculated between outcome variables (Table 2). All variables showed statistically significant correlations, except for age and climate change stressors. Effect sizes (*r*^2^, “shared variance”) were mostly moderate to large. Correlations between age and stressor groups were low. As indicated by the 95% confidence intervals, the relationships between the Covid-19 stressors and all three SSQ scores showed statistically higher correlations than the relationships with the two other stressor groups.

**Table 2:** Gender differences and zero-order correlations between aga and all main outcome variables

variable	gender		variable					
	*M* (*SD)* (male)	*M* (*SD)* (female)	2. SSQ sum	3. SSQ psycho-logical	4. SSQ physical	5. Covid-19 pandemic stressors	6. Ukraine crisis stressors	7. climate change stressors
1. age	50.6 (17.5)	50.3 (16.8)	*r* = -.34 [-.37—-.31] *p* < .001	*r* = -.37 [-.40—-.34] *p* < .001	*r* = -.25 [-.27—-.20] *p* < .001	*r* = -.11 [-.15—-.08] *p* < .001	*r* = .12 [.09—.16] *p* < .001	*r* < -01 [-.04—.04] *p* = .981
2. SSQ sum	23.6 (19.7)	28.7 (21.3)		*r* = .98 [.97—.98] *p* < .001	*r* = .90 [.90—.91] *p* < .001	*r* = .55 [.52—.57] *p* < .001	*r* = .30 [.27—.34] *p* < .001	*r* = .32 [.29—.35] *p* < .001
3. SSQ psychological	16.0 (13.5)	19.5 (14.7)			*r* = .79 [.78—.81] *p* < .001	*r* = .53 [.50—.56] *p* < .001	*r* = .29 [.25—.32] *p* < .001	*r* = .31 [.27—.34] *p* < .001
4. SSQ physical	7.6 (7.3)	9.2 (7.7)				*r* = .51 [.48—.53] *p* < .001	*r* = .28 [.25—.31] *p* < .001	*r* = .30 [.27—.33] *p* < .001
5. Covid-19 pandemic stressors	1.6 (.9)	1.9 (.9)					*r* = .60 [.57—.62] *p* < .001	*r* = .52 [.49—.55] *p* < .001
6. Ukraine crisis stressors	2.2 (.9)	2.5 (.9)						*r* = .66 [.64—.68] *p* < .001
7. climate change stressors	1.7 (1.0)	2.0 (1.0)						-

*Abbreviations: SSQ* Subclinical Stress Questionnaire, *M* Mean, *SD* Standard Deviation. *r* Spearman correlation coefficients. *95%* confidence intervals are displayed in squared brackets. *p* = level of significance

### Gender-differences across sub-clinical stress and stressor scale scores

Differences between male and female participants were calculated. Although male participants scored lower on all variables (SSQ sum: *U* = 1,029,016.5, *Z* = 6.74, *p* < 0.001, *η* = 0.12; SSQ psychological: *U* = 1,031,668.0, *Z* = 6.64, *p* < 0.001, *η* = 0.12; SSQ physical: *U* = 1,049,763.0, *Z* = 5.92, *p* < 0.001, *η* = 0.11; Covid-19 pandemic stressors: *U* = 978,200.0, *Z* = 8.80, *p* < 0.001, *η* = 0.16; Ukraine crisis stressors: *U* = 919,160.5, *Z* = 11.18, *p* < 0.001, *η* = 0.21; climate change stressors: *U* = 992,839.0, *Z* = 8.22, *p* < 0.001, *η* = 0.15), effect sizes were too small to be considered meaningful.

### Differences in rankings between stressor scales

**Fig. 1:**
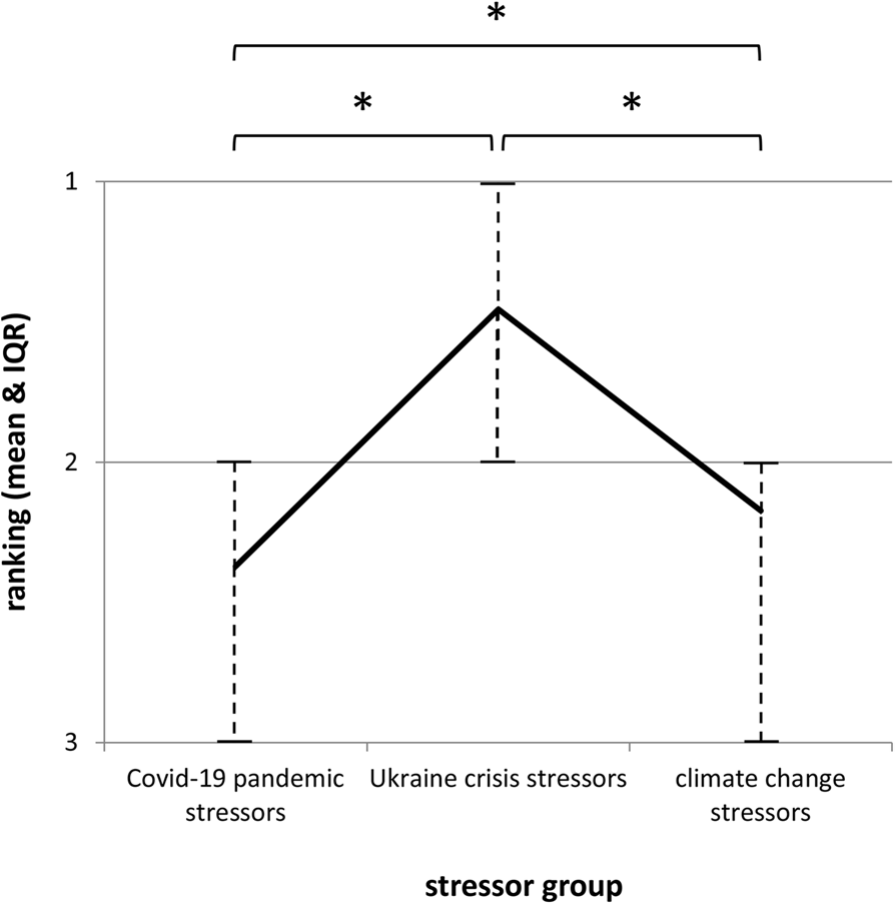
Mean ranks (paired samples) and interquartile ranges for the three stress groups. Note. Asterisks indicate statistically significant pairwise comparisons on a *p* < .001 – level

Figure 1 displays the mean ranks for the three stressor groups, as well as the interquartile range (IQR). Individuals prioritized worries related to the stressor groups significantly different (*Chi*^2^ (2) = 1541.50, *p* < 0.001; *W* = 0.25). All pairwise comparisons were statistically significant too (climate change stressors – Ukraine crisis stressors: *Z* = 29.65; p < 0.001; *r* = 0.53; climate change stressors – Covid-19 pandemic stressors: *Z* = 8.27; *p* < 0.001; *r* = 0.15; Ukraine crisis stressors – Covid 19-pandemic stressors: *Z* = 33.94; *p* < 0.001; *r* = 0.61). In summary, Ukraine crisis stressors were rated as the most significant stressor group (mean rank: 1.46; IQR: 1 – 2), followed by climate change stressors (mean rank: 2.17; IQR: 2 – 3), and Covid-19 pandemic stressors (mean rank: 2.37; IQR: 2 – 3).

### Predicting subclinical stress from stressor scales, age, and gender

Table 3 displays the full and the final multiple linear regression model for the prediction of the SSQ sum score, including standardized beta-values as well as 95% confidence intervals. The final model fitted the data (*F*(5, 3088) = 393.16, *p* < 0.001, maximum VIF = 1.45, may cook’s *d* = 0.01). As indicated by the significant gender effect, female respondents experienced higher stress symptoms in general. The significant interaction implies a less significant impact of Covid-19 stressors on stress symptoms with increasing age. Ukraine crisis stressors were excluded from the final model, as they had no statistically significant impact on the SSQ sum score, when controlling for other stressor types. The Covid-19 stressors main effect had the highest effect size. Stress symptoms also increased with worries about climate related stressors, but only with a low effect size. The final model also fitted the two SSQ subscales (psychological (*F*(5, 3088) = 381.54, *p* < 0.001, maximum VIF = 1.45, may cook’s *d* = 0.02, *R*^*2*^ = 0.38) and physical subclinical stress symptoms (*F*(5, 3088) = 267.04, *p* < 0.001, maximum VIF = 1.45, may cook’s *d* = 0.01, *R*^*2*^ = 0.30)), except that the gender effect was no longer a significant predictor in the latter model.

**Table 3:** Multivariate linear regression models for the prediction of the SSQ sum score for the full model and final model after stepwise backward exclusion

*Predictor*	model							
	***full model***				***final model***			
	*standardized β*	95% CI low	95% CI up		*standardized β*	95% CI low	95% CI up	
***Sociodemographic variables***								
age	**-.30**	**-.33**	**-.27**	** < .001**	**-.30**	**-.33**	**-.27**	** < .001**
gender	**-.04**	**-.07**	**-.01**	**.010**	**-.04**	**-.07**	**-.01**	**.010**
***Stressor groups***								
Covid-19	**.48**	**.44**	**.52**	** < .001**	**.48**	**.44**	**.51**	** < .001**
Ukraine	< -.01	-.05	.04	.861				
Climate	**.06**	**.02**	**.10**	**.004**	**.06**	**.02**	**.09**	**.001**
***Interactions***								
Interaction age * climate change stressors	-.03	-.07	.01	.129				
Interaction age * Ukraine crisis stressors	.01	-.03	.05	.676				
Interaction age * Covid-19 pandemic stressors	**-.05**	**-.09**	**-.02**	**.003**	**-.07**	**-.09**	**-.04**	** < .001**
Interaction gender * climate change stressors	.01	-.05	.03	.692				
Interaction gender * Ukraine crisis stressors	-.03	-.07	.01	.125				
Interaction gender * Covid-19 pandemic stressors	.04	-.01	.07	.055				
***R***^***2***^	**.39**			** < .001**	**.39**			** < .001**

*Abbreviations: R*^2^ Proportion of explained variance, *CI* Confidence intervals, *low* lower bound, *up* upper bound. *p* = level of significance

## Discussion

This study aimed to disentangle the worries related to the three recent population stressors (climate change, Covid-19 pandemic, and Ukraine crisis) from its impact on current subclinical stress symptoms. As a major finding, the most recent Ukraine crisis ranked number one as the most worrying stressor at the time of assessment. However, only the Covid-19 related stressors were significantly related to stress symptoms with a reasonable effect size when controlling for other stressors in the immediate aftermath of the fifth Covid-19 wave in Germany.

One evident explanation for the obtained results lies in the temporal proximity of the stressors: Longitudinal research has demonstrated that the existence of the Covid-10 pandemic per se is not stressful, but that the stress it exerts varies by the different phases of it [48, 49]. The sampling took place in close temporal proximity to the fifth Covid-19 wave in Germany. Thus, the direct consequences of the pandemic, which are associated with adverse mental health effects, such as social distancing [50, 51], direct health consequences [52, 53], or everyday activitiesx [54, 55] might have been among the directly perceivable consequences of the Covid-19 pandemic. Another explanation may refer to trauma research: Stressful events are associated with stress reactions when directly addressing aspects of our sense of coherence [25, 56], for example one's sense of control, security, or predictability of events. This loss of a sense of control and its relation to Covid-19 related health effects has also been already discussed in recent publications [57, 58]: The threat is omnipresent as soon as one enters the public. In addition, the virus as a potential aggressor cannot directly be encountered and effective treatment options were sparse in the beginning. For the absence of an effect of the Ukraine war -though generating intense compassion for war victims, strongly focused by media coverage- it could we proposed that this population stressor possesses no threat to one’s own security and the sense of control in everyday life, in terms of the proximity of the stressor. Moreover, research on the impact of an individual’s locus of control on stress demonstrates that compassion and the ability to grant support (internal locus of control) is associated with better stress-regulation abilities [59–61]. This is also supported by researcher that demonstrates differences in risk perception and the people’s actions in relation to the Covid-19 pandemic and climate change, which may apply to the war too [62]. Thus, it could be speculated that Germans who might develop stress symptoms related to the Ukraine crisis might buffer this risk by for example actively engaging into aid activities. The present study does not provide data on this manner but could guide future investigations in this field. The climate crisis in turn could be similarly abstract regarding the feeling of subjective threat. Although its medium-term threats to our way of life are well known, the concrete effects are probably not yet sufficiently noticeable (apart from individuals who had directly experienced events such as the recent flood disaster in north-western/south Germany).

An alternative explanation may address the temporal duration of events that becomes necessary to generate stress symptoms: This would be consistent with findings of physiological stress research indicating that chronic stress exposure leads to maladaptation and a variety of stress-associated symptoms at the physiological level due to prolonged cortisol exposure [63]. The Covid-19 pandemic has already been affecting society for more than two years, whereas the war in Ukraine may not have lasted long enough to generate pronounced stress responses yet. This would mean that with continued duration, this war could also generate stress and associated symptoms. However, this contrasts with the current discussion of a so-called "war fatigue" that seems to be spreading among the German population, and which has already described in the context of other violent conflicts [8]. This phenomenon is associated with the “compassion fatigue”, an emotional, physical, and social exhaustion that overcomes a person when continually confronted with the suffering of others, leading to a profound decline in their ability to empathize compassionately with others [64]. This phenomenon is sufficiently described among professionals working in crisis regions [65]. It is conceivable that a similar compassion fatigue in the sense of a non-clinically relevant habituation to terrifying images from the media may also occur within the general population that is not directly exposed to the events war. Initially, people may feel a strong compassion with the war victims due to dramatic images of media coverage, followed by habituation. Research on adverse effects of the Covid-19 pandemic has also demonstrated that some personally traits may be advantageous as they help individuals to distance themselves from the potential stressors and adapt to daily hassles [66]. The climate crisis, finally, has been going on for a long time, but the sense of concrete threat has probably not yet reached the population. This is in line with latest research, suggesting that people in the global south experience climate change stressors more directly, while populations that do not suffer direct consequences may experience a collective state of denial [52]. Taken together, it may be precisely the combination of long duration and the threat to the sense of coherence and security that leads to a measurable stress response in people due to the COVID-19 pandemic. A longitudinal follow-up assessment would shed light on the differential effects of those potential factors.

Regarding specific vulnerable subgroups, older individuals and male respondents reported less and/or less severe stress symptoms, and an older age further decreased the impact of Covid-19 stressors on subclinical stress symptoms. Our findings are in line with previous research of other groups [27]. In a recent U.S. sample, younger age, female sex, and caregiver status were associated with higher levels of stress response to the Covid-19 pandemic [44]. The same findings revealed a recent Chinese study: Although the exact mechanisms were unclear, authors supposed that older persons may direct more cognitive effort to maintaining positive effects, or that younger individuals might experience greater responsibilities to their and younger generations. Women’s higher stress, on the other hand, was explained with research suggesting that women are generally at higher risk for mental health outcomes [67]. Further, higher psychological stress in women may be partly explained by their work being professionally closer to Covid-19 victims and the care burden in home [68]. To investigate this hypothesis, future research should include measures on factors that help to shed light on specific mechanisms in this subgroup.

The present study also faces some limitations: One limiting parameter was its online format. Although the age range also covered older respondents, limited access to mobile devices could have been a barrier for participation. Likewise, individuals with severe health deficits might have been prevented from participation. Furthermore, the cross-sectional design is only correlational in nature. The composition of stressor scales could also not be based on an exhaustive and systematic review of public media.

## Conclusions

In summary, it is conceivable that a measurable stress response particularly occurs if several parameters of stressful events co-occur. Special preventive attention should be paid to young people and women because they show more pronounced stress reactions. Taken together, the present study disentangled the impact of three major population stressors currently affecting Germany, as a high-income western European country. It sheds light on the significance of different stressors and has the potential to inform future studies that target etiological risk factors and health trajectories not only for future clinically significant psychiatric and associated disorders from a contemporary perspective, but also for minor impacts on psychosocial wellbeing and functioning in the aftermath of crises. In particular, it is suggested that researchers and practitioners in the field of global health and prevention do not only consider the worries people have about certain population stressors, but that they in particular acknowledge the special and temporal proximity of those stressors.

## Data Availability

The datasets used and/or analysed during the current study are available from the corresponding author on reasonable request.
